# Foodborne Titanium Dioxide Nanoparticles Aggravated Secondary Liver Injury in DSS-Induced Colitis: Role of the NLRP3 Inflammasome

**DOI:** 10.3390/foods14183279

**Published:** 2025-09-22

**Authors:** Xiaoyan Feng, Hongbin Yuan, Tao You, Hengyi Xu

**Affiliations:** State Key Laboratory of Food Science and Resource, Nanchang University, Nanchang 330047, China; 357900210014@email.ncu.edu.cn (X.F.); 18807045301@163.com (H.Y.); 407900210112@email.ncu.edu.cn (T.Y.)

**Keywords:** titanium dioxide nanoparticles, secondary liver injury, inflammatory response, NLRP3 inflammasome

## Abstract

Secondary liver injury (SLI) is the most common complication in the development of inflammatory bowel disease (IBD), and it is susceptible to environmental factors, including diet patterns. As a food-brightening agent, titanium dioxide nanoparticles (TiO_2_ NPs) are inevitably consumed by IBD patients. Currently, there are a few studies on TiO_2_ NPs exposure to SLI in colitis mice. In this study, a SLI model was built using dextran sodium sulfate (DSS) free-drinking for 7 days after pre-exposure to TiO_2_ NPs. The changes in the pathological results and liver function indicators suggested that high-dose TiO_2_ NPs only exhibited a slight injury in the liver. With further analysis, we found that pre-exposure to high-dose TiO_2_ NPs in mice with SLI led to an increase in intestinal permeability and hepatic LPS content, along with increased inflammatory cytokines and an anti-oxidative system imbalance. Subsequently, accumulated LPS and ROS overproduction activated the NOD-like receptor family pyrin-containing 3 (NLRP3) inflammasome, inducing hepatic cell pyroptosis. To provide compelling evidence, NLRP3 gene-deficient mice were used, and the results showed that the absence of NLRP3 improved liver function, alleviated hepatic inflammation, and reduced hepatic oxidative injury in SLI mice with TiO_2_ NPs exposure. In summary, these results confirmed the critical role of the NLRP3 inflammasome in the TiO_2_ NP-aggravated progression of SLI. Our study provided a comprehensive evaluation of foodborne nanoparticles on IBD complications, hoping that more studies can focus on IBD complications affected by environmental factors.

## 1. Introduction

Inflammatory bowel disease (IBD) is characterized as chronic, recurrent, and non-specific intestinal inflammation, including ulcerative colitis (UC) and Crohn’s disease (CD) [1]. Globally, more than 6.8 million patients suffer from IBD, and the morbidity of IBD worldwide is still rising [2,3], especially in newly industrialized countries in Asia, Africa, and South America [4]. It is extremely important that there are multiple extraintestinal complications in the development of IBD, and these complications are the main reason for the increased difficulty of further treatment and health risks [5,6], which significantly results in the increased morbidity and mortality of IBD. Noteworthily, severe extraintestinal manifestations of IBD are hepatobiliary abnormalities—approximately 70% of the blood supply is from the portal vein. Indeed, the gastrointestinal tract is anatomically closely linked with the hepatobiliary system by the mesenteric and portal vein, so the liver is naturally the first and direct target of colonic inflammatory responses in IBD patients [7,8]. It has been reported that 5–10% of IBD patients could develop secondary liver diseases, including fatty liver, autoimmune hepatitis, and cirrhosis. Mendes et al. [9] found that 27% of patients with UC showed liver biochemical abnormalities. Now, many researchers believe that secondary liver injury (SLI), a well-known complication in IBD patients, is significantly related to an over-inflammation response [10]. For IBD patients, the intestinal barrier is damaged (showing increased intestinal permeability, decreased intestinal mucosa, etc.), resulting in various microorganisms and its metabolites (e.g., LPS), and inflammatory cytokines (e.g., TNF-α, IL-1β) are transmitted to the liver across the intestinal barrier, resulting in liver inflammation, which further accelerates the development of UC and eventually leads to a vicious circle [11,12].

Environmental changes, including diet patterns, contamination, smoking, etc., are an important contributor to the development of extraintestinal manifestations in IBD patients [13]. Although IBD patients and the healthy population share the same environment, IBD patients are more susceptible to external substances, which may cause more severe health issues [14]. Wang et al. [15] found that 6:2 chloro-polyfluorooctane ether sulfonate (F-53B) administration significantly increased inflammatory cytokine levels and slowed the self-healing of UC mice. Luo et al. [16] investigated the adverse effect of PS-MPs exposure to dextran sodium sulfate (DSS)-induced colitis, suggesting that alone, PS-MPs gavage caused minimal effects in the intestinal barrier, while a shorter colon length, an exacerbated inflammatory response, and increased intestinal permeability were found in DSS-induced colitis mice with PS-MPs exposure. Moreso, Yang et al. [17] gavaged triclocarban (an antimicrobial ingredient and environmental contaminants) to colitis mice and found that triclocarban exaggerated colonic inflammation and colitis-associated colon tumorigenesis. Therefore, more research should give attention to the body’s response when IBD patients are exposed to exogenous substances.

Titanium dioxide (TiO_2_) is a natural non-silicate mineral oxide and exists in different forms [18]. TiO_2_ is usually used as a food additive, and its main function is as a brightening agent, namely E171 [19,20]. Notably, more than 36% of food-grade TiO_2_ belongs to nano-sized particles (<100 nm), and it has been reported that large amounts of titanium dioxide nanoparticles (TiO_2_ NPs) were found in candy, gum, etc. [21,22]. In fact, food-grade TiO_2_ NPs exhibit no effect or a low toxicity on distal organs, characterized by low-grade inflammation, oxidative injury, and cell apoptosis, and are unable to cause serious illness [23]. With the increasing application of TiO_2_ particles in food, IBD patients are inevitably exposed to them, as confirmed by Lomer et al., who evaluated the dietary intake of TiO_2_ NPs in CD patients and healthy volunteers, and found that a median daily intake of TiO_2_ NPs consumed by patients was 2.5 mg [24]. Now, the risk assessment of TiO_2_ NPs exposure in IBD has been conducted. The administration of TiO_2_ NPs induced more severe intestinal injury in colitis mice via NOD-like receptor family pyrin-containing 3 (NLRP3) overactivation and ROS excessive production [25,26]. Urrutia-Ortega et al. [27] administered 5 mg/kg BW E171 to mice with colitis-associated cancer (CAC), and the results showed that only E171 exposure was unable to induce tumor formation; however, increased tumor formation in the distal colon was observed in the E171-exposure CAC model. All the above research reported that TiO_2_ NPs can worsen pre-existing intestinal disease, but a few studies focused on the impact of TiO_2_ NPs on SLI in mice.

As the primary detoxification organ, the liver is a direct extraintestinal target that is affected by colitis. Therefore, the impact of TiO_2_ NPs exposure on SLI in colitis mice cannot be ignored. Based on these considerations, we explored the impact and potential mechanisms of TiO_2_ NPs exposure on DSS-induced SLI. In this study, the histopathological structure, liver function, inflammatory cytokine levels, and the extent of oxidative injury were evaluated to illustrate that TiO_2_ NPs have a negative effect on DSS-induced SLI. Importantly, we found that the inflammatory response was involved in TiO_2_ NP-aggravated SLI in mice. To provide further evidence, we confirmed the NLRP3 inflammasome’s crucial role in TiO_2_ NP-aggravated SLI by virtue of gene-deficient mice. Our study aims to understand the effects of foodborne nanoparticles on extraintestinal organs in IBD patients, which could provide a potential therapeutic target for multifactorial IBD.

## 2. Materials and Methods

### 2.1. Animal Experiment and Design

Male wildtype (WT) C57BL/6J mice (6–8 weeks) were purchased from SiPeiFu Biotechnology Co., Ltd. (Certificate No. SCXK (Jing) 2019–0010, Beijing, China). NLRP3-deficient (NLRP3^−/−^) C57BL/6J mice (6–8 weeks) were purchased from Wuhan Youdu Biotechnology Co., Ltd. (Certificate No. SCXK (e) 2021−0025, Wuhan, China). Male and female mice were mated, and their offspring (male mice) were used in this experiment. All mice were housed at 25 °C with 12 h light/12 h dark cycles and a humidity of 50 ± 5% and were acclimated for 1 week. Meanwhile, all mice were allowed free access to food and water ad libitum during the entire experimental period. All animal experiments strictly adhered to the Guidelines for Care and Use of Laboratory Animals as well as the ARRIVE guidelines and were conducted with ethical approval from the Institutional Animal Care Committee (IACUC) of Nanchang Royo Biotech Co., Ltd. (IACUC Number: RYE2024061104, Nanchang, China). Interventional drug preparations and coding were conducted by a third person who was not involved in this experiment so as to ensure blinding. Moreover, all animals were housed on the same shelf in the same room to minimize potential confounders. Importantly, all animals were handled humanely to reduce their suffering, and euthanasia of the mice was carried out by cervical dislocation after CO_2_ anesthesia.

All mice were randomly assigned to receive different treatments (n = 6/group). For TiO_2_ NPs exposure (the TiO_2_ NPs group), mice were gavaged with 2 mg/kg BW/d (low-dose) and 20 mg/kg BW/d (high-dose) TiO_2_ NPs, respectively. For the DSS-induced SLI model (the SLI group), mice were given free access to 3.5% DSS (m/v) drinking water for 7 consecutive days. For TiO_2_ NPs exposure in SLI mice (the SLI + TiO_2_ NPs group), mice were pre-exposed to 2 mg/kg BW/d or 20 mg/kg BW/d TiO_2_ NP exposure for 6 weeks, followed by 3.5% DSS free-drinking for 7 days during the 6^th^ week. The same size (n = 6/group) was determined based on previously similar studies with sufficient statistical power to detect differences in the primary endpoints. TiO_2_ NPs (Shanghai Aladdin Biochemical Technology Co., Ltd., CAS: 13463-67-7, Shanghai, China) were dispersed in sterile deionized water and underwent ultrasound for 30 min to make a uniform suspension. At the end of the experiment, all mice were sacrificed, and then their blood and liver were collected for subsequent analyses.

### 2.2. Histopathological Analysis

After all the mice were sacrificed, fresh liver tissues were fixed in paraformaldehyde overnight, paraffinized, and sectioned at a thickness of 5 μm. Subsequently, these slices were stained with hematoxylin and eosin (H&E). Images were captured using an optical microscope (Olympus Corporation, Tokyo, Japan), and inflammatory cell infiltration was observed in the liver.

### 2.3. Liver Function Assessment

Fresh blood was collected and placed at 4 °C for 6 h and then centrifuged for 15 min to obtain serum samples. Next, serum AKP and AST activities were used to assess liver function and were measured using commercial biochemical kits according to the operating instructions (Nanjing Jiancheng Bioengineering Institute, Nanjing, China).

### 2.4. Liver Inflammation and Oxidative Injury

Liver tissues were homogenized with normal saline to prepare the samples for subsequent analysis. Inflammatory cytokines, including TNF-α, IL-6, and IL-1β, were measured following the commercial ELISA kit’s instructions (Shanghai Yansheng Industrial Co., Ltd., Shanghai, China), and OD values at 450 nm were determined by a microplate reader (Thermo Fisher Scientific, Waltham, MA, USA). In addition, SOD activity, MDA content, and GSH content were chosen as indicators for the assessment of oxidative injury in the liver, and these were detected using commercial kits (Nanjing Jiancheng Bioengineering Institute, Nanjing, China).

### 2.5. Real-Time Quantitative Polymerase Chain Reaction (RT-qPCR) Analysis

Fresh liver tissues were preserved at −80 °C for subsequent mRNA expression analysis. Total RNA was extracted using Trelief^®^ RNAprep FastPure Tissue & Cell Kit (TSINGKE Biotechnology Co., Ltd., Beijing, China), and total RNA concentrations were quantified using the BioDrop μLite + ultra-micro spectrophotometer (Harvard Bioscience Company, Holliston, UK). Subsequently, complementary DNA was reversed transcribed by using the Hifair^®^ Ⅲ 1st Strand cDNA Synthesis Supermix (Yeasen Biotechnology Co., Ltd., Shanghai, China). Finally, RT-qPCR programs were prepared by using the 2 × TSINGKE Master qPCR Mix (SYBR Green Ⅰ with UDG) (TSINGKE Biotechnology Co., Ltd., Beijing, China) and performed using the Agilent AriaMx Real-time PCR Program (Agilent Technologies, Santa Clara, CA, USA). The relative mRNA expressions were determined by using the 2^−ΔΔt^ method, and β-actin was used for normalization. The sequence information of the primers is listed in Table 1.

### 2.6. Statistical Analysis

All graphs were plotted using GraphPad Prism 8 software (GraphPad Software Inc., USA). Statistical analyses were performed by using SPSS 27, and the intergroup differences were determined using one-way analysis of variance (ANOVA) followed by the LSD multiple comparison test. All data were represented as the mean ± standard error of mean (SEM), and a *p* < 0.05 was considered statistically significant.

## 3. Results

### 3.1. TiO_2_ NPs Aggravated the Symptoms of DSS-Induced SLI

**Figure 1:**
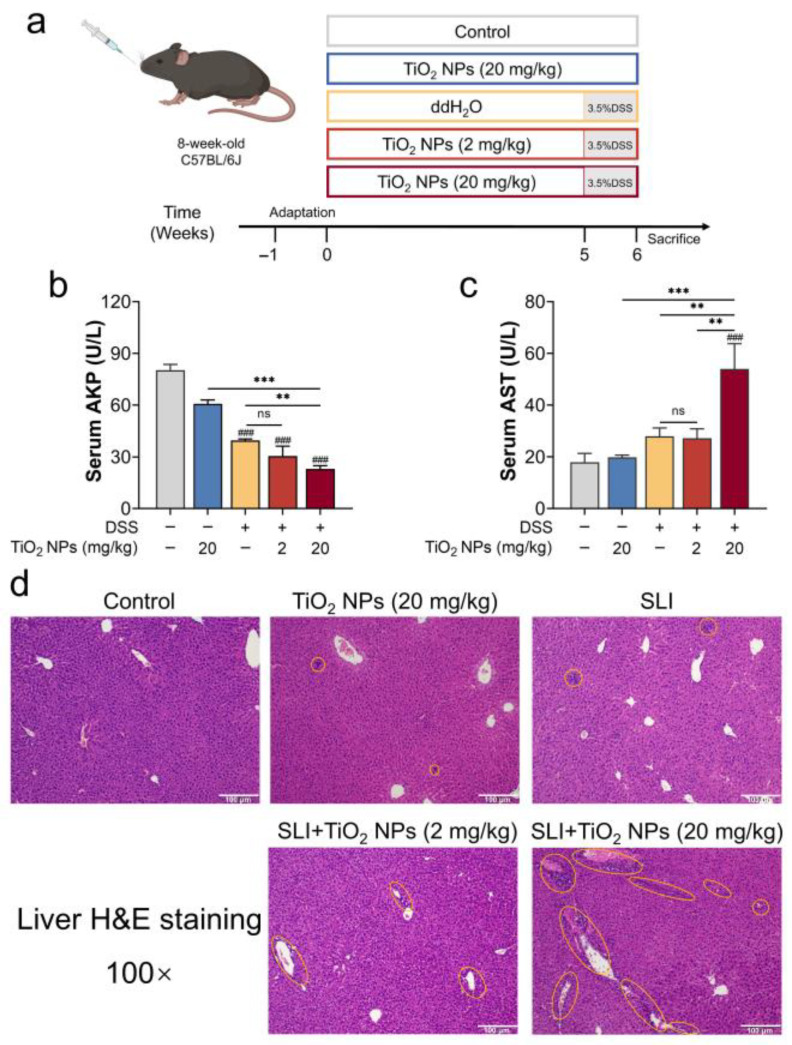
Titanium dioxide nanoparticles (TiO_2_ NPs) affected liver function in dextran sodium sulfate (DSS)-induced secondary liver injury (SLI). (**a**) Schematic diagram of animal experiment design. Liver function indicators: (**b**) AKP activity and (**c**) AST activity in serum. (**d**) Representative hematoxylin and eosin (H&E) images of liver, in which the yellow ring indicates inflammatory cell infiltration; scale bar: 100 μm. Data are represented as the mean ± SEM (n ≥ 3). Compared with the control group, ### *p* < 0.001; ** *p* < 0.01, *** *p* < 0.001, * indicates significant intergroup differences; “ns” indicates no statistically significant difference.

Firstly, the effects of exposure to TiO_2_ NPs on SLI in mice were evaluated (Figure 1a). As liver function indicators, serum AKP and AST activities were measured, and the results are shown in Figure 1b,c. Compared with the SLI group, no significant differences in serum AKP and AST activities were observed in the SLI + L-TiO_2_ NPs group, while high-dose TiO_2_ NPs significantly increased serum AST activity and decreased serum AKP activity in SLI mice. Moreover, histopathological H&E images are shown in Figure 1d; the yellow ring indicates inflammatory cell infiltration, and its size corresponds to the extent of inflammatory response. Obviously, hepatocytes were orderly arranged and plump in the control group. Regardless of the high-dose TiO_2_ NPs given to the exposure group, the SLI model group only showed mild inflammatory cell infiltration. Notably, exposure to TiO_2_ NPs enhanced inflammatory cell infiltration in SLI, in which exposure to high-dose TiO_2_ NPs caused more severe inflammatory cell infiltration than low-dose TiO_2_ NPs. These results preliminarily confirmed that TiO_2_ NPs can impair liver function in SLI mice, and that high-dose TiO_2_ NPs caused more severe liver injury.

### 3.2. TiO_2_ NPs Induced Liver Inflammation and Oxidative Injury in DSS-Induced SLI Mice

**Figure 2:**
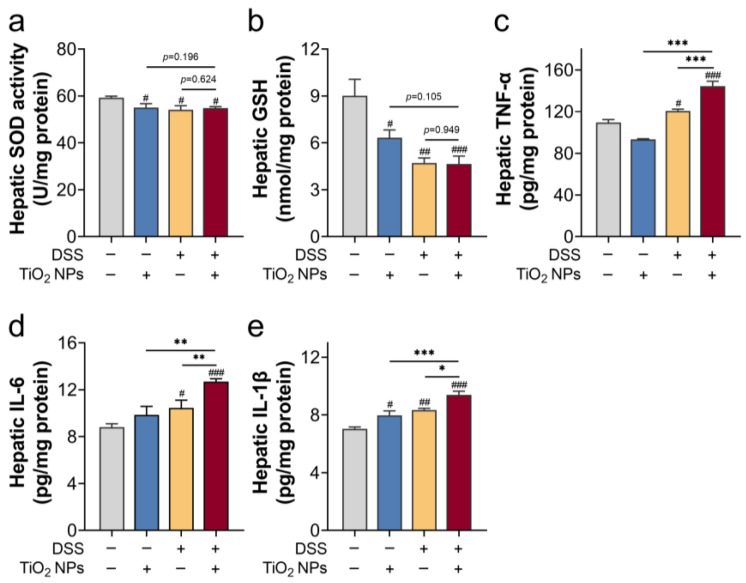
Comprehensive evaluation of hepatic inflammation and oxidative injury after TiO_2_ NPs exposure in SLI mice. (**a**) Hepatic SOD activity, (**b**) hepatic GSH content, (**c**) hepatic TNF-α content, (**d**) hepatic IL-6 content, and (**e**) hepatic IL-1β content. Data are represented as the mean ± SEM (n ≥ 3). Compared with the control group, # *p* < 0.05, ## *p* < 0.01, ### *p* < 0.001; * *p* < 0.05, ** *p* < 0.01, *** *p* < 0.001, * indicates significant intergroup differences.

From the perspective of histopathological analysis and liver function evaluation, we found that exposure to high-dose TiO_2_ NPs induced severe symptoms in SLI mice. Next, hepatic inflammation and oxidative injury were assessed (Figure 2). In Figure 2a,b, a significant decrease in SOD activity and GSH content suggested that TiO_2_ NPs and DSS exposure may induce hepatic oxidative injury. Still, there was no significant difference between the TiO_2_ NPs group/SLI group and the SLI + TiO_2_ NPs group (*p* > 0.05). In Figure 2c–e, exposure to high-dose TiO_2_ NPs enhanced the inflammatory response in SLI mice. Collectively, compared with the TiO_2_ NPs exposure group/SLI group, no significant increase in oxidative injury was found in the TiO_2_ NP-aggravated SLI, in which the inflammatory response contributed to the TiO_2_ NP-aggravated SLI in mice.

### 3.3. The NLRP3 Inflammasome Was Probably Involved in TiO_2_ NP-Aggravated SLI

**Figure 3:**
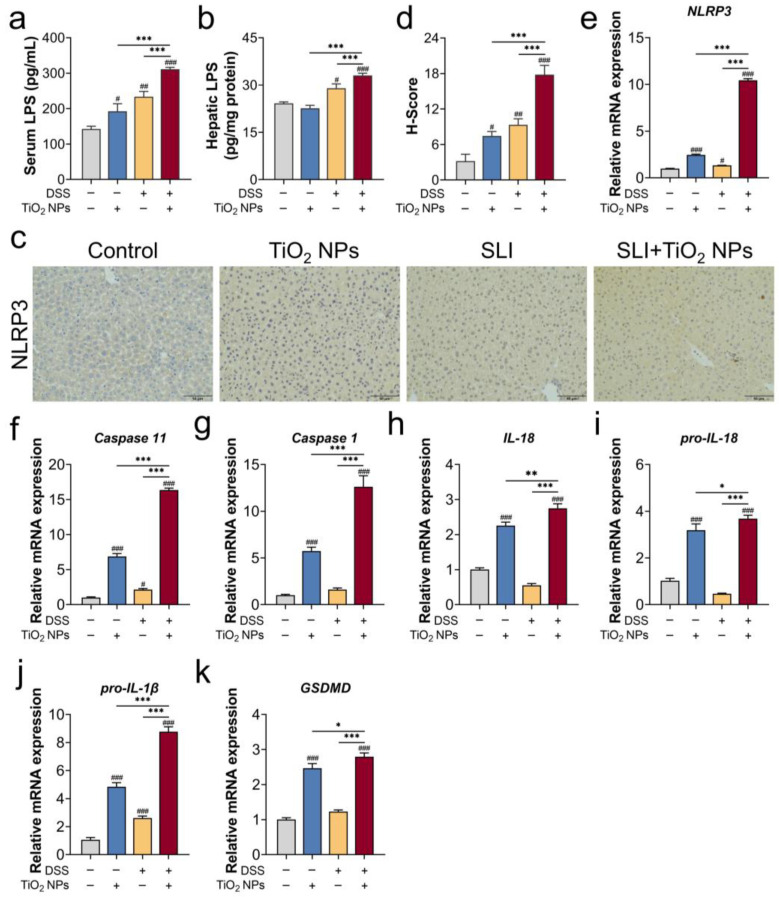
The NOD-like receptor family pyrin-containing 3 (NLRP3) inflammasome may be involved in TiO_2_ NP-aggravated SLI in mice. (**a**) Serum LPS, (**b**) hepatic LPS, (**c**) representative immunohistochemistry images, and (**d**) quantitative analysis of NLRP3 protein expression. H-Score = 3 × high positive + 2 × positive + 1 × low positive; scale bar: 50 μm. (**e**–**k**) Relative mRNA expression of the NLRP3 inflammasome-related genes using RT-qPCR analysis. Data are represented as the mean ± SEM (n ≥ 3). Compared with the Control group, # *p* < 0.05, ## *p* < 0.01, ### *p* < 0.001; * *p* < 0.05, ** *p* < 0.01, *** *p* < 0.001, * indicates significant intergroup differences.

An inflammatory response that contributed to TiO_2_ NP-aggravated SLI in mice was confirmed, and the specific mechanism requires further exploration. Firstly, the serum LPS contents were measured (Figure 3a). Compared with the TiO_2_ NPs exposure group and the SLI model group, theserum LPS contents sharply rose in the SLI + TiO_2_ NPs group, indicating an increase in intestinal permeability. Importantly, once the intestinal barrier is destroyed, exogenous substances and pathogen-derived toxins (e.g., LPS) can be transferred into the bloodstream from the intestine. In Figure 3b, the hepatic LPS content in the SLI + TiO_2_ NPs group exhibited an abnormal increase, suggesting that LPS was transferred into the blood and accumulated in the liver, thereby exacerbating liver injury. Furthermore, the expression of the NLRP3 protein and NLRP3 inflammasome-related genes (e.g., Caspase 11, Caspase 1, IL-18) was significantly upregulated in the SLI + TiO_2_ NPs group (Figure 3c–k). In summary, hepatic LPS activated the NLRP3 inflammasome, thereby causing more severe liver injury in SLI mice exposed to TiO_2_ NPs.

### 3.4. The Absence of NLRP3 Alleviated the Symptoms of DSS-Induced SLI with TiO_2_ NPs Exposure

**Figure 4:**
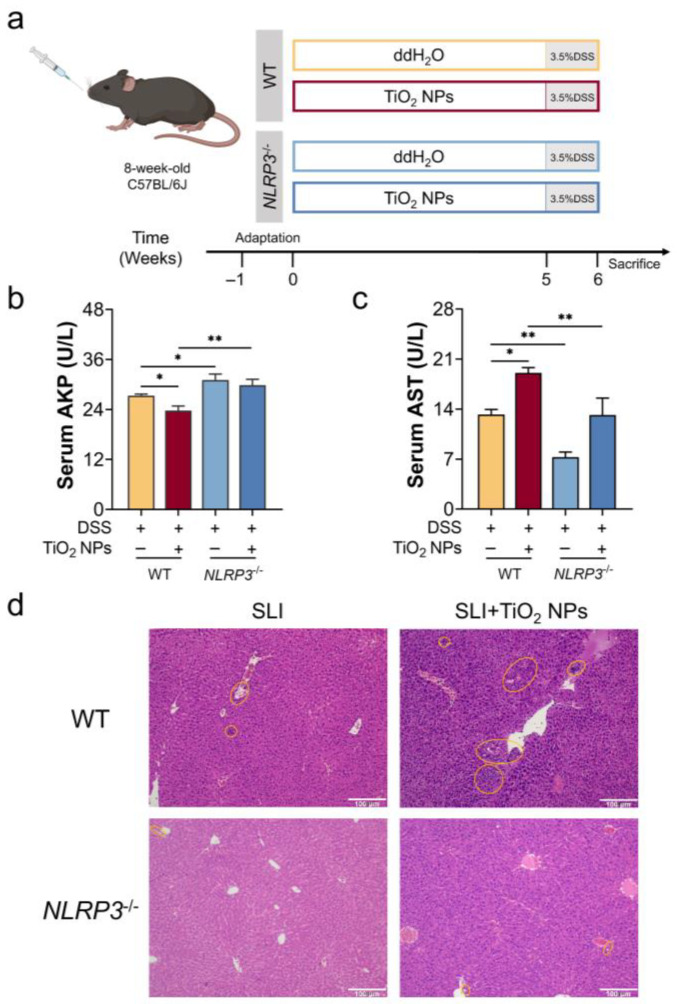
NLRP3 deficiency improved liver function in TiO_2_ NP-aggravated SLI in mice. (**a**) Schematic diagram of animal experiment design. (**b**) Serum AKP activity and (**c**) serum AST activity as indicators for liver function assessment. (**d**) Representative H&E images of liver tissue, in which the yellow ring indicates inflammatory cell infiltration; scale bar: 100 μm. Data are represented as the mean ± SEM (n ≥ 3). * *p* < 0.05, ** *p* < 0.01, * indicates significant intergroup differences.

To provide compelling evidence, NLRP3^−/−^ mice were utilized to fully demonstrate the critical role of the NLRP3 inflammasome in TiO_2_ NP-aggravated SLI in mice (Figure 4a). As shown in Figure 4b,c, exposure to TiO_2_ NPs induced a decrease in serum AKP activity and an increase in AST activity in SLI mice, which was reversed by the absence of NLRP3. In addition, liver tissues were stained with H&E, and the acquired images are shown in Figure 4d. Compared with the SLI group, hepatic sections in the SLI + TiO_2_ NPs group exhibited an extensive inflammatory cell infiltration, while attenuated liver injury was observed in the NLRP3^−/−^ mice. These results agreed that the absence of NLRP3 would improve liver function in DSS-induced SLI after exposure to TiO_2_ NPs.

### 3.5. The Absence of NLRP3 Mitigated Liver Injury in SLI Mice with TiO_2_ NPs Exposure

**Figure 5:**
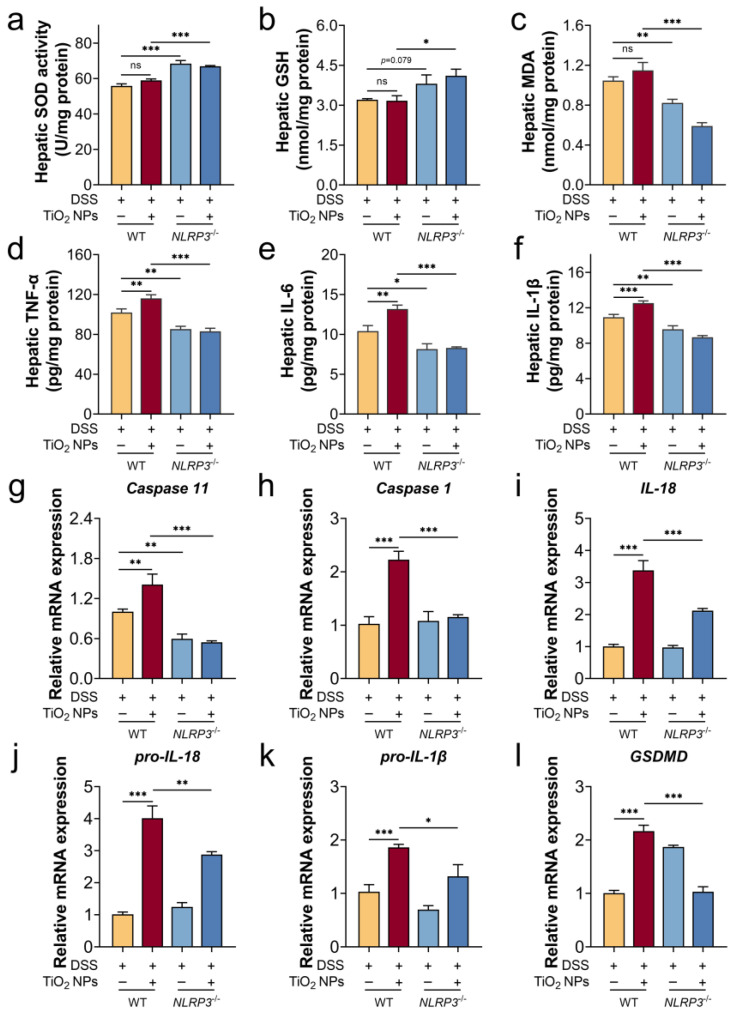
TiO_2_ NPs triggered the overactivation of NLRP3 inflammasome to aggravate DSS-induced SLI. Oxidative levels: (**a**) hepatic SOD activity, (**b**) hepatic GSH content, and (**c**) hepatic MDA content. Inflammatory cytokine levels: (**d**) hepatic TNF-α content, (**e**) hepatic IL-6 content, and (**f**) hepatic IL-1β content. (**g**–**l**) Relative mRNA expression of the NLRP3 inflammasome-related genes. Data are represented as the mean ± SEM (n ≥ 3). * *p* < 0.05, ** *p* < 0.01, *** *p* < 0.001; ns indicates no statistically significant difference; * indicates significant intergroup differences.

We gavaged high-dose TiO_2_ NPs daily to NLRP3^−/−^ mice with DSS-induced colitis and found that the absence of NLRP3 improved the anti-oxidative ability of the liver (Figure 5a–c), which was manifested by a decreased MDA content, enhanced SOD activity, and increased GSH content. Meanwhile, inflammatory cytokine (TNF-α, IL-6, and IL-1β) levels were detected (Figure 5d–f), and there was a significant reduction in the hepatic inflammatory levels in the NLRP3^−/−^ mice. An in-depth quantitative analysis of NLRP3 inflammasome-related gene expression was performed using RT-qPCR. In Figure 5g–l, exposure to TiO_2_ NPs exacerbated the progression of the SLI mice via NLRP3 inflammasome overactivation, which was supported by a significant downregulation of the NLRP3 inflammasome-related genes in the NLRP3^−/−^ mice, including Caspase 11, Caspase 1, IL-18, pro-IL-18, and pro-IL-1β. Therefore, the absence of NLRP3 improved the anti-oxidative ability, reduced the inflammatory response, and ultimately mitigated the TiO_2_ NP-exacerbated progression of SLI.

## 4. Discussion

With the acceleration of modern industrialization, IBD has become a global health concern [4]. As a food additive, TiO_2_ NPs are usually added to candy, jam, pastries, etc. Now, growing evidence has elucidated that TiO_2_ NPs accelerate the intestinal progression of IBD [26,27]. Importantly, the complications of IBD are also non-negligible for us. As a detoxification organ, the liver is a direct extraintestinal target of colonic inflammatory responses in IBD [7,8]. Currently, insufficient investigations have explored the impact of TiO_2_ NPs exposure on SLI in IBD. In this study, we focused on delineating how exposure to TiO_2_ NPs affects SLI in mice and further elucidated its underlying mechanisms.

TiO_2_ NPs ingestion is unavoidable at any age, so we consider that TiO_2_ NPs pre-exposure to mice may replicate the real exposure scenarios for the human body. Our results showed that exposure to 20 mg/kg TiO_2_ NPs did not cause obvious hepatic abnormality in mice (Figure 1). A study showed that doses of 0, 2, 10, and 50 mg/kg/d TiO_2_ NPs were gavaged for 90 consecutive days; however, there were no remarkable changes in serum ALT and AST activities, although exposure to 50 mg/kg TiO_2_ NPs induced mild oxidative injury [28]. Thus, the selected high-dose TiO_2_ NPs were deemed to cause low toxicity to the liver. Additionally, the adverse effects of pre-exposure to TiO_2_ NPs on SLI were assessed. In accordance with previous research [29,30], we built an SLI model by DSS-drinking for 7 consecutive days. According to the results of H&E staining as well as the pro-inflammatory cytokines (Figure 1 and Figure 2c–e), after DSS free-drinking, the inflammatory response became elevated in the liver. Under the “inflammatory burden”, the body may be more susceptible to TiO_2_ NPs; therefore, we conclude that pre-exposure to TiO_2_ NPs accelerated the progression of SLI in mice.

LPS is an endotoxin produced by Gram-negative bacteria, and this resides in the intestinal lumen if the intestinal barrier is intact. Once the intestinal barrier is damaged, LPS is translocated into the bloodstream, crossing the intestinal barrier. Therefore, elevated LPS levels in serum imply intestinal barrier dysfunction. As our results show, exposure to TiO_2_ NPs increased the LPS contents in serum. Similarly, Zhu et al. [31] found that long-term exposure to TiO_2_ NPs downregulated the expression of the MUC2 gene and absorbed mucin protein to induce the intestinal mucus layer disruption. Yan et al. [32] gavaged mice with doses of 10, 40, and 160 mg/kg BW TiO_2_ NPs exposure for 28 days, demonstrating that regardless of the dosages, exposure to TiO_2_ NPs reduced the intestinal mucus thickness and decreased the expression of the MUC2 gene. Therefore, long-term pre-exposure to TiO_2_ NPs compromised intestinal barrier integrity. Herein, we used DSS to build an SLI model. Substantial evidence has demonstrated that DSS disrupted intestinal barrier dysfunction in the DSS-induced model, including intestinal mucosal barrier dysfunction, the proliferation inhibition of intestinal epithelial cells [33], and, in addition, the serum LPS levels increased [34]. In short, pre-exposure to TiO_2_ NPs disrupted the intestinal barrier, further leading to increased intestinal permeability in DSS-induced SLI, which was supported by the presented results (Figure 3a). The gastrointestinal tract is anatomically closely linked to the liver by the portal vein, so serum LPS was further transferred and accumulated in the liver, as confirmed by our results (Figure 3b). That is to say, LPS serves as a mediator of the aggravated liver injury.

NLRP3 inflammasome, a protein complex, belongs to the innate immune system and is activated by two pathways, including the noncanonical and canonical NLRP3 signals [35], and its activation triggers cell pyroptosis. The canonical NLRP3 inflammasome depends on the self-cleavage of pro-caspase 1 and the maturation of IL-1β and IL-18. Dissimilarly, the noncanonical NLRP3 inflammasome activation is initiated by intracellular LPS via direct interactions with caspase 11, inducing autoproteolysis and the activation of caspase 11 [36,37,38]. Hence, accumulated LPS triggered noncanonical NLRP3 overactivation in the liver. Using a general perspective, ROS overproduction has been implicated in canonical NLRP3 inflammasome activation in different ways, such as ROS-sensed thioredoxin-interacting protein [39,40] and the MAPK/NF-κB pathway [41]. Our results showed that TiO_2_ NPs mainly induced a moderate oxidative injury, thereby activating the canonical NLRP3 inflammasome. Unexpectedly, hepatic LPS was undetected in the TiO_2_ NPs exposure group, but relative mRNA expression of the caspase 11 gene was upregulated in the liver (Figure 3f), suggesting that the noncanonical NLRP3 inflammasome may be independent of LPS stimulation. As explained by Lupfer et al. [42], ROS directly upregulated the expression of Caspase 11, followed by activating the noncanonical NLRP3 inflammasome via JNK signaling. Additionally, abnormal inflammation in the liver significantly increased in the SLI + TiO_2_ NPs group, and the exacerbation effect of hepatic ROS and LPS was attributed to the synergic activation of the NLRP3 inflammasome (Figure 3). Meanwhile, NLRP3 gene-knockout experiments provided direct evidence for the pivotal role of the NLRP3 inflammasome in TiO_2_ NP-aggravated SLI (Figure 4 and Figure 5).

Our work focused on the adverse effect of pre-exposure to TiO_2_ NPs on SLI, further providing convincing evidence to clarify that NLRP3 inflammasome overactivation was involved in the aggravated effect. This study provides a theoretical basis for an in-depth understanding of foodborne nanoparticles on extraintestinal organs in IBD. Although our findings provide new insight, further investigations are also needed in this study. In future studies, we can try to use different models to jointly verify the important role of the NLRP3 inflammasome. Moreover, we can also explore whether the NLRP3 inflammasome plays a critical role in long-term exposure to TiO_2_ NPs in the chronic SLI model.

## 5. Conclusions

In our study, pre-exposure to high-dose TiO_2_ NPs showed only a minimal impact on the liver. However, pre-exposure to TiO_2_ NPs had an adverse effect on DSS-induced SLI, mainly manifested as an anti-oxidative system imbalance and abnormal inflammation. Severe liver injury was attributed to increased intestinal permeability, thereby hepatic LPS and ROS synergistically activated the NLRP3 inflammasome signal, finally inducing hepatic cell pyroptosis. Our work aims to better understand the critical role of exposure to foodborne nanoparticles in the progression of extraintestinal manifestations in IBD patients and provides an intervention strategy for multifactorial IBD that is affected by environmental factors.

## Figures and Tables

**Table 1:** The primer sequences of RT-qPCR analysis.

Gene	Primer	Sequence (5′-3′)
NLRP3	Forward	ATTACCCGCCCGAGAAAGG
	Reverse	CATGAGTGTGGCTAGATCCAAG
Caspase 11	Forward	GTGGTGAAAGAGGAGCTTACAGC
	Reverse	GCACCAGGAATGTGCTGTCTGA
Caspase 1	Forward	GGCACATTTCCAGGACTGACTG
	Reverse	GCAAGACGTGTACGAGTGGTTG
IL-18	Forward	GACAGCCTGTGTTCGAGGATATG
	Reverse	TGTTCTTACAGGAGAGGGTAGAC
pro-IL-18	Forward	GACTCTTGCGTCAACTTCAAGG
	Reverse	CAGGCTGTCTTTTGTCAACGA
GSDMD	Forward	TGTCAACCTGTCAATCAAGGA
	Reverse	AGCCAAAACACTCCGGTTC
pro-IL-1β	Forward	GCAACTGTTCCTGAACTCAACT
	Reverse	ATCTTTTGGGGTCCGTCAACT
β-actin	Forward	AAGGCCAACCGTGAAAAGAT
	Reverse	GTGGTACGACCAGAGGCATAC

## Data Availability

The original contributions presented in the study are included in the article, further inquiries can be directed to the corresponding author.

## References

[1] Boccatonda A., Balletta M., Vicari S., Hoxha A., Simioni P., Campello E. (2022). The journey through the pathogenesis and treatment of venous thromboembolism in inflammatory bowel diseases: A narrative review. Semin. Thromb. Hemost..

[2] Liu C., Qi X., Liu X., Sun Y., Mao K., Shen G., Ma Y., Li Q. (2024). Anti-inflammatory probiotics HF05 and HF06 synergistically alleviate ulcerative colitis and secondary liver injury. Food Funct..

[3] Meng Z., Fu B., Yang Z., Xu Y., Huang H., Bai Y., Fang X., Shen S., Yang J., Yong J. (2023). Polydopamine-coated thalidomide nanocrystals promote DSS-induced murine colitis recovery through Macrophage M2 polarization together with the synergistic anti-inflammatory and anti-angiogenic effects. Int. J. Pharm..

[4] Ng S., Shi H., Hamidi N., Underwood F., Tang W., Benchimol E., Panaccione R., Ghosh S., Wu J., Chan F. (2017). Worldwide incidence and prevalence of inflammatory bowel disease in the 21st century: A systematic review of population-based studies. Lancet.

[5] Zhu L., Zong X., Xiao X., Cheng Y., Fu J., Lu Z., Jin M., Wang F., Wang Y. (2022). Multi-omics analysis of the gut-liver axis reveals the mechanism of liver injury in colitis mice. Front. Immunol..

[6] Silva J., Brito B., Silva I., Nóbrega V., Silva M., Gomes H., Fortes F., Pimentel A., Mota J., Almeida N. (2019). Frequency of hepatobiliary manifestations and concomitant liver disease in inflammatory bowel disease patients. BioMed. Res. Int..

[7] Trivedi P., Jena G. (2013). Ulcerative colitis-induced hepatic damage in mice: Studies on inflammation, fibrosis, oxidative DNA damage and GST-P expression. Chem-Biol. Interact..

[8] Lama S., Pagano E., Borrelli F., Maisto M., Tenore G., Nanì M., Chacon-Millan P., Novellino E., Stiuso P. (2024). Polyphenol-rich extract from ‘Limoncella’ apple variety ameliorates dinitrobenzene sulfonic acid-induced colitis and linked liver damage. Int. J. Mol. Sci..

[9] Mendes F., Levy C., Enders F., Loftus E., Angulo P., Lindor K. (2007). Abnormal hepatic biochemistries in patients with inflammatory bowel disease. Off. J. Am. Coll. Gastroenterol..

[10] Li E., Wang T., Zhou R., Zhou Z., Zhang C., Wu W., He K. (2021). Myricetin and myricetrin alleviate liver and colon damage in a chronic colitis mice model: Effects on tight junction and intestinal microbiota. J. Funct. Foods..

[11] Chen Y., Zhu S., Chen Z., Liu Y., Pei C., Huang H., Hou S., Ning W., Liang J. (2022). Gingerenone A alleviates ferroptosis in secondary liver injury in colitis mice via activating Nrf2–Gpx4 signaling pathway. J. Agric. Food Chem..

[12] Chen Y., Lu Y., Pei C., Liang J., Ding P., Chen S., Hou S. (2020). Monotropein alleviates secondary liver injury in chronic colitis by regulating TLR4/NF-κB signaling and NLRP3 inflammasome. Eur. J. Pharmacol..

[13] Sandys O., te Velde A. (2022). Raising the alarm: Environmental factors in the onset and maintenance of chronic (low-grade) inflammation in the gastrointestinal tract. Digest. Dis. Sci..

[14] Zheng H., Wang J., Wei X., Chang L., Liu S. (2021). Proinflammatory properties and lipid disturbance of polystyrene microplastics in the livers of mice with acute colitis. Sci. Total Environ..

[15] Wang C., Pan Z., Jin Y. (2023). F-53B induces hepatotoxic effects and slows self-healing in ulcerative colitis in mice. Environ. Pollut..

[16] Luo T., Wang D., Zhao Y., Li X., Yang G., Jin Y. (2022). Polystyrene microplastics exacerbate experimental colitis in mice tightly associated with the occurrence of hepatic inflammation. Sci. Total. Environ..

[17] Yang H., Sanidad K., Wang W., Xie M., Gu M., Cao X., Xiao H., Zhang G. (2019). Triclocarban exposure exaggerates colitis and colon tumorigenesis: Roles of gut microbiota involved. Gut Microbes.

[18] Candás-Zapico S., Kutscher D., Montes-Bayón M., Bettmer J. (2018). Single particle analysis of TiO_2_ in candy products using triple quadrupole ICP-MS. Talanta.

[19] Peters R., van Bemmel G., Herrera-Rivera Z., Helsper H., Marvin H., Weigel S., Tromp P., Oomen A., Rietveld A., Bouwmeester H. (2014). Characterization of titanium dioxide nanoparticles in food products: Analytical methods to define nanoparticles. J. Agric. Food Chem..

[20] Cui Y., Gong X., Duan Y., Li N., Hu R., Liu H., Hong M., Zhou M., Wang L., Wang H. (2010). Hepatocyte apoptosis and its molecular mechanisms in mice caused by titanium dioxide nanoparticles. J. Hazard. Mater..

[21] Weir A., Westerhoff P., Fabricius L., Hristovski K., von Goetz N. (2012). Titanium dioxide nanoparticles in food and personal care products. Environ. Sci. Technol..

[22] Baranowska-Wójcik E., Szwajgier D., Winiarska-Mieczan A. (2022). A review of research on the impact of E171/TiO_2_ NPs on the digestive tract. J. Trace Elem. Med. Biol..

[23] Zhao Y., Liu S., Tang Y., You T., Xu H. (2021). *Lactobacillus rhamnosus* GG ameliorated long-term exposure to TiO_2_ nanoparticles induced microbiota-mediated liver and colon inflammation and fructose-caused metabolic abnormality in metabolism syndrome mice. J. Agric. Food Chem..

[24] Lomer M., Hutchinson C., Volkert S., Greenfield S., Catterall A., Thompson R., Powell J. (2004). Dietary sources of inorganic microparticles and their intake in healthy subjects and patients with Crohn’s disease. Br. J. Nutr..

[25] Ruiz P., Moron B., Becker H., Lang S., Atrott K., Spalinger M., Scharl M., Wojtal K., Fischbeck-Terhalle A., Frey-Wagner I. (2017). Titanium dioxide nanoparticles exacerbate DSS-induced colitis: Role of the NLRP3 inflammasome. Gut.

[26] Duan S., Wang H., Gao Y., Wang X., Lyu L., Wang Y. (2023). Oral intake of titanium dioxide nanoparticles affect the course and prognosis of ulcerative colitis in mice: Involvement of the ROS-TXNIP-NLRP3 inflammasome pathway. Part. Fibre Toxicol..

[27] Urrutia-Ortega I., Garduño-Balderas L., Delgado-Buenrostro N., Freyre-Fonseca V., Flores-Flores J., González-Robles A., Pedraza-Chaverri J., Hernández-Pando R., Rodríguez-Sosa M., León-Cabrera S. (2016). Food-grade titanium dioxide exposure exacerbates tumor formation in colitis associated cancer model. Food Chem. Toxicol..

[28] Chen Z., Zhou D., Han S., Zhou S., Jia G. (2019). Hepatotoxicity and the role of the gut-liver axis in rats after oral administration of titanium dioxide nanoparticles. Part. Fibre Toxicol..

[29] Duan S., Du X., Chen S., Liang J., Huang S., Hou S., Gao J., Ding P. (2020). Effect of vitexin on alleviating liver inflammation in a dextran sulfate sodium (DSS)-induced colitis model. Biomed. Pharmacother..

[30] Wang Q., Liu Y., Gao L., Zhang L., Wang J. (2024). Study on the protective effect and mechanism of umbilicaria esculenta polysaccharide in DSS-induced mice colitis and secondary liver injury. J. Agric. Food Chem..

[31] Zhu X., Zhao L., Liu Z., Zhou Q., Zhu Y., Zhao Y., Yang X. (2020). Long-term exposure to titanium dioxide nanoparticles promotes diet-induced obesity through exacerbating intestinal mucus layer damage and microbiota dysbiosis. Nano Res..

[32] Yan J., Wang D., Li K., Chen Q., Lai W., Tian L., Lin B., Tan Y., Liu X., Xi Z. (2020). Toxic effects of the food additives titanium dioxide and silica on the murine intestinal tract: Mechanisms related to intestinal barrier dysfunction involved by gut microbiota. Environ. Toxicol. Phar..

[33] Zhang C., He A., Liu S., He Q., Luo Y., He Z., Chen Y., Tao A., Yan J. (2019). Inhibition of HtrA2 alleviated dextran sulfate sodium (DSS)-induced colitis by preventing necroptosis of intestinal epithelial cells. Cell Death. Dis..

[34] Jiang N., Wei Y., Cen Y., Shan L., Zhang Z., Yu P., Wang Y., Xu L. (2020). Andrographolide derivative AL-1 reduces intestinal permeability in dextran sulfate sodium (DSS)-induced mice colitis model. Life Sci..

[35] Faria S., Costantini S., de Lima V., de Andrade V., Rialland M., Cedric R., Budillon A., Magalhães K. (2021). NLRP3 inflammasome-mediated cytokine production and pyroptosis cell death in breast cancer. J. Biomed. Sci..

[36] Moretti J., Jia B., Hutchins Z., Roy S., Yip H., Wu J., Shan M., Jaffrey S., Coers J., Blander J. (2022). Caspase-11 interaction with NLRP3 potentiates the noncanonical activation of the NLRP3 inflammasome. Nat. Immunol..

[37] Matikainen S., Nyman T., Cypryk W. (2020). Function and regulation of noncanonical caspase-4/5/11 inflammasome. J. Immunol..

[38] Huang Y., Xu W., Zhou R. (2021). NLRP3 inflammasome activation and cell death. Cell Mol. Immunol..

[39] Tschopp J., Schroder K. (2010). NLRP3 inflammasome activation: The convergence of multiple signalling pathways on ROS production?. Nat. Rev. Immunol..

[40] Zhou J., Chng W. (2013). Roles of thioredoxin binding protein (TXNIP) in oxidative stress, apoptosis and cancer. Mitochondrion.

[41] Li Z., Chi H., Zhu W., Yang G., Song J., Mo L., Zhang Y., Deng Y., Xu F., Yang J. (2021). Cadmium induces renal inflammation by activating the NLRP3 inflammasome through ROS/MAPK/NF-κB pathway in vitro and in vivo. Arch. Toxicol..

[42] Lupfer C., Anand P., Liu Z., Stokes K., Vogel P., Lamkanfi M., Kanneganti T. (2014). Reactive oxygen species regulate caspase-11 expression and activation of the non-canonical NLRP3 inflammasome during enteric pathogen infection. PLoS Pathog..

